# Role of DNA methylation on the association between physical activity and cardiovascular diseases: results from the longitudinal multi-ethnic study of atherosclerosis (MESA) cohort

**DOI:** 10.1186/s12864-021-08108-w

**Published:** 2021-11-03

**Authors:** Hangchuan Shi, Deborah J. Ossip, Nicole L. Mayo, Daniel A. Lopez, Robert C. Block, Wendy S. Post, Alain G. Bertoni, Jingzhong Ding, Si Chen, Chen Yan, Zidian Xie, Ina Hoeschele, Yongmei Liu, Dongmei Li

**Affiliations:** 1 Department of Clinical and Translational Research, University of Rochester Medical Center, Rochester, NY 14642-0708 USA; 2 Department of Public Health Sciences, University of Rochester Medical Center, Rochester, NY 14642 USA; 3 Division of Cardiology, Department of Medicine, Johns Hopkins University School of Medicine, Baltimore, MD 21287 USA; 4 Division of Public Health Sciences, Wake Forest School of Medicine, Winston-Salem, NC 27157 USA; 5 Department of Medicine, Wake Forest Baptist Medical Center, Winston-Salem, NC 27157 USA; 6 Aab Cardiovascular Research Institute, University of Rochester, School of Medicine and Dentistry, Rochester, NY 14642 USA; 7 Department of Pharmacology and Physiology, University of Rochester, School of Medicine and Dentistry, Rochester, NY 14642 USA; 8 Department of Statistics, Fralin Life Sciences Institute at Virginia Tech, Blacksburg, VA 24061 USA; 9 Department of Medicine, Division of Cardiology, Duke Molecular Physiology Institute, Duke University Medical Center, Durham, NC 27701 USA

**Keywords:** Cardiovascular disease, Physical activity, DNA methylation, MESA, Structural equation modeling

## Abstract

**Background:**

The complexity of physical activity (PA) and DNA methylation interaction in the development of cardiovascular disease (CVD) is rarely simultaneously investigated in one study. We examined the role of DNA methylation on the association between PA and CVD.

**Results:**

The Multi-Ethnic Study of Atherosclerosis (MESA) cohort Exam 5 data with 1065 participants free of CVD were used for final analysis. The quartile categorical total PA variable was created by activity intensity (METs/week). During a median follow-up of 4.0 years, 69 participants developed CVD. Illumina HumanMethylation450 BeadChip was used to provide genome-wide DNA methylation profiles in purified human monocytes (CD14+). We identified 23 candidate DNA methylation loci to be associated with both PA and CVD. We used the structural equation modeling (SEM) approach to test the complex relationships among multiple variables and the roles of mediators. Three of the 23 identified loci (corresponding to genes *VPS13D*, *PIK3CD* and *VPS45*) remained as significant mediators in the final SEM model along with other covariates. Bridged by the three genes, the 2nd PA quartile (β = − 0.959; 95%CI: − 1.554 to − 0.449) and the 3rd PA quartile (β = − 0.944; 95%CI: − 1.628 to − 0.413) showed the greatest inverse associations with CVD development, while the 4th PA quartile had a relatively weaker inverse association (β = − 0.355; 95%CI: − 0.713 to − 0.124).

**Conclusions:**

The current study is among the first to simultaneously examine the relationships among PA, DNA methylation, and CVD in a large cohort with long-term exposure. We identified three DNA methylation loci bridged the association between PA and CVD. The function of the identified genes warrants further investigation in the pathogenesis of CVD.

**Supplementary Information:**

The online version contains supplementary material available at 10.1186/s12864-021-08108-w.

## Background

Cardiovascular disease (CVD) is the leading cause of mortality in the United States [1]. Understanding contributors to the etiology of CVD will help us better control the disease at the level of primary prevention [2]. The etiology of CVD involves complex multifactorial mechanisms including environmental and genetic factors [3]. Among the environmental or behavioral exposures, physical activity (PA) has consistently been shown in epidemiological studies to be associated with reduced risk of CVD [4]. Although multiple mediating mechanisms have been proposed (e.g., an anti-inflammatory response, insulin-sensitivity) [4, 5], our knowledge about the underlying mechanisms of the benefits of PA on cardiovascular health is still sparse. Owing to the evolutionary development of genomic study methods, recent studies suggest that epigenetic modifications are also involved in the association between PA and CVD [6].

DNA methylation is the best-studied epigenetic modification. It is a dynamic epigenetic mechanism in several cellular process such as regulating gene expression, genomic imprinting, and X-chromosome inactivation [7]. In eukaryotes, the methylated cytosine (5-methylcytosine, 5mC) occurs predominantly at C followed by guanine (G) forming the so-called CpG site. Although the result of DNA methylation is usually linked to the silencing of genes [8], growing evidences showed that DNA methylation can also increase gene expression in some instances [9]. Epigenome-wide association studies (EWAS) have shown that many DNA methylation loci are associated with CVD traits [10, 11] and influenced by physical activity [12]. However, because the associations between PA or CVD and DNA methylation were studied separately in previous studies, the role of DNA methylation on PA’s benefits on cardiovascular health was only deduced from the biological functions of identified genes [13]. There is a lack of evidence directly showing the complexity of PA-DNA methylation interaction in the development of CVD [14].

To investigate the complex relationships among PA, DNA methylation, and CVD, we applied the structural equation modeling (SEM) approach to a cohort. Compared with common statistical methodologies, SEM allows for testing of complex relationships among multiple variables including the roles of mediators [15]. In epigenetic epidemiology, it is useful to investigate whether DNA methylation is a component of the pathway linking the exposure and the outcome [14]. In the current study, we evaluated the role of DNA methylation on the longitudinal association between PA and CVD, along with other covariates, using data from the Multi-Ethnic Study of Atherosclerosis (MESA).

## Methods

### Data source and study cohort

The MESA study is a prospective cohort study designed to investigate the prevalence, associations, and progression of subclinical CVD in 6814 men and women aged 45–84 years and free of clinical CVD at the first examination (Exam 1, 2000–2002). Following Exam 1, five follow-up examinations (Exam 2–6) have occurred. Written informed consent was obtained from all participating subjects under research protocols approved by the review boards of the six filed centers. A detailed description of the MESA study be found elsewhere [16]. Corresponding to Exam 5 (2010–2012), the MESA Epigenomics and Transcriptomics Study (2010–2012) was conducted to investigate the association between DNA methylation and gene expression in purified human monocytes (CD14^+^) [17]. Asian participants were not included since the study was not available for participants from two of six MESA sites [18]. The DNA methylation data are publicly accessible through the GEO website with accession ID GSE56046.

In this study, we linked the MESA DNA methylation data to Exam 5 data through unique MESA participant IDs. As a result, we used exposure (physical activity), covariate, and DNA methylation data of 1264 participants collected in 2010–2012 (Exam 5). Data were analyzed and compared for CVD outcomes occurring through December 31, 2015. We excluded participants with prevalent CVD at baseline (*n* = 155) and those with missing exposure or covariate data (*n* = 44). The final sample size of the analyzed cohort was 1065.

### Variables and analysis

#### Physical activity

Physical activity and sedentary behaviors were assessed through an interviewer-administered MESA Typical Week Physical Activity Survey (TWPAS) which was designed to estimate participants’ habitual activities as of their last interview. The survey contains 28 question item categories asking participants about the duration of time when doing each category of activities. The unit of the continuous activity variables was measured as both minutes per week and metabolic equivalence of tasks (METs) per week. According to the MET value of each activity, the intensity was classified according to MET value as sedentary behavior (METs ≤1.5), light PA (1.5 < METs < 3.0), moderate PA (3 ≤ METs < 6), and vigorous PA (METs ≥6) [19, 20]. In the screening stage of this study, the continuous total PA variable was defined as the sum of total METs per week spent doing light, moderate, and vigorous PAs. The quartile categorical total PA variable (METs/week) was created from the continuous total PA variable used both in screening stage and in the SEM model to examine potential non-linear pattern. In addition to total PA, each PA components (i.e., light, moderate, and vigorous PA), two combinations of PA intensity (i.e., moderate to vigorous PA [MVPA] and exercise PA), and leisure sedentary behavior were also used for screening PA related DNA methylation loci to increase the screening power. The detailed descriptions of all PA variables are listed in Supplementary Table S1.

#### CVD outcomes

Each MESA participant was contacted every 9–12 months to collect information on new CVD conditions, which were classified using medical records and death certificates [16]. MESA assessed 11 individual cardiovascular events. The detailed descriptions of all CVD outcomes are listed in Supplementary Table S2. In this study, CVD (all types), which was the combination of all 11 individual cardiovascular events, was used as the CVD outcome in the SEM model. The endpoint outcome was the binary response (Yes/No) to CVD (all types) occurring through December 31, 2015. However, in the screening stage of DNA methylation loci identification, all individual CVD outcomes were used separately to increase the sensitivity of screening.

#### Covariates

Atherosclerotic CVD (ASCVD) 10-year risk score is developed by the American College of Cardiology (ACC) and American Heart Association (AHA) in collaboration with the National Heart Lung and Blood Institute (NHLBI). The score is in a percentage form to estimate the risk of a hard ASCVD event within the next ten years [21, 22]. The risk estimator is calculated based on an individual’s sex, race, age, total cholesterol (mg/dL), high-density lipoprotein cholesterol (HDL, mg/dL), systolic blood pressure, use of blood pressure medication, smoking status, and diabetes status. The calculation additionally takes into account the potential interactions between these variables.

Some potential confounders associated with CVD in the literature [23–25], but absent in the ASCVD score, included ethnicity (Hispanic or not), body mass index (BMI), waist circumference (WC, cm), and coronary artery calcium (CAC) score. BMI and WC were categorized into three categories based on well-established definitions [26]. CAC score is the quantification of the coronary artery calcification by computed tomography (CT) [27]. In this study, we used the Agatston CAC score and categorized it into four levels (0, 1–100, 101–400, > 400) according to one of the most widely used classification systems [28].

#### Identification of DNA methylation locus

Illumina HumanMethylation450 (450 K) BeadChip was used to provide genome-wide DNA methylation profiles with over 485,000 DNA methylation loci. Quality control was descripted in detail in a previous study [18]. Prior to identification, CpG sites were filtered according to beta values (cut-off = 0.2) to exclude background changes [29]. A two-stage strategy was then used. In the first stage (screening stage), EWAS were performed using *limma* and *preprocessCore* packages in R [30, 31]. Separate univariate logistic regression models were fitted using the normalized M-value for each CpG site as the dependent variable. Each of the PA related variables (continuous or categorical) or each of the CVD outcome variables (binary) was used as the independent variable. *P*-values were adjusted for multiple testing using the Benjamini-Hochberg method for controlling the false discovery rate (FDR), which was discussed in our previous publications [32, 33]. CpG sites with adjusted *p*-value < 0.05 were selected as potential candidates. All candidate CpG sites screened from regression models with PA related variables were pooled as Set A, and all candidate CpG sites from models with CVD outcome variables were pooled as Set B. In the second stage (identification stage), CpG sites overlapped by Set A and Set B were finally identified as the final candidate DNA methylation loci for the further SEM model. The corresponding genes were determined using Illumina annotation. Volcano plots and heatmaps were created using *ggplot2* and *pheatmap* packages in R [34, 35] to show the differentially methylated level on the identified DNA loci.

#### SEM model

SEM analyses were performed to examine the proposed conceptual model, namely, the role of DNA methylation on the association between PA and CVD. Model fits were conducted using Mplus version 8.5 (Muthen and Muthen, 2017). All the parameters in the SEM model were estimated by a maximum likelihood method [36]. The estimated coefficient (a.k.a. coefficient load, β) generated from the SEM model was used to measure the strength of association between two variables. Prior to model fitting, the correlation matrix of all identified DNA methylation loci was examined based on M-values to avoid multicollinearity. We did SEM model selection by including the categorical total PA variable (MET-mins/week), CVD (all), and all identified DNA methylation loci, along with all covariates. Association was considered non-significant if the coefficient load β had a *p*-value > 0.1. Variables lacking significant associations with other variables were removed from the SEM model. The final SEM model was fitted using variables with at least one significant direct or indirect path from PA to CVD. Sensitivity analyses were conducted by fitting SEM models and substituting the continuous PA variable for the categorical PA variable or excluding mediators from the final SEM model.

## Results

### Demographic and clinical characteristics of the studied samples

During a median of 4.0 years’ follow-up, 69 of 1065 participants had a cardiovascular disease event. The cumulative incidence is 6.5%. The baseline characteristics of the 1065 participants revealed an average age of 69.2 years (range: 54–92 years) with a slightly higher proportion of females (52.3%) than males (Table 1). The largest group of the participants were non-Hispanic White (46.9%), followed by Hispanics (31.3%) and non-Hispanic Blacks (21.8%). Compared with participants who did not develop any CVD event (non-incident cases), incident cases were more likely to be older people (72.7 years vs 69.0 years), to have hypertension (71.0% vs 57.0%), and undergoing hypertension treatment (69.6% vs 52.4%). Notably, compared with non-incident cases, incident cases were more likely to be identified in the lowest PA quartile (39.1% vs 24.4%), but less likely to be in the second (17.4% vs 25.5%) or third PA quartile (15.9% vs 25.5%). At baseline, the mean ASCVD (atherosclerotic cardiovascular disease) score of the future incident cases was higher (28.5%) than that of non-incident cases (19.3%). Also, incident cases were more likely to have a CAC (coronary artery calcium) score greater than 100 (55.0%) than non-incident cases (33.6%).

**Table 1:**
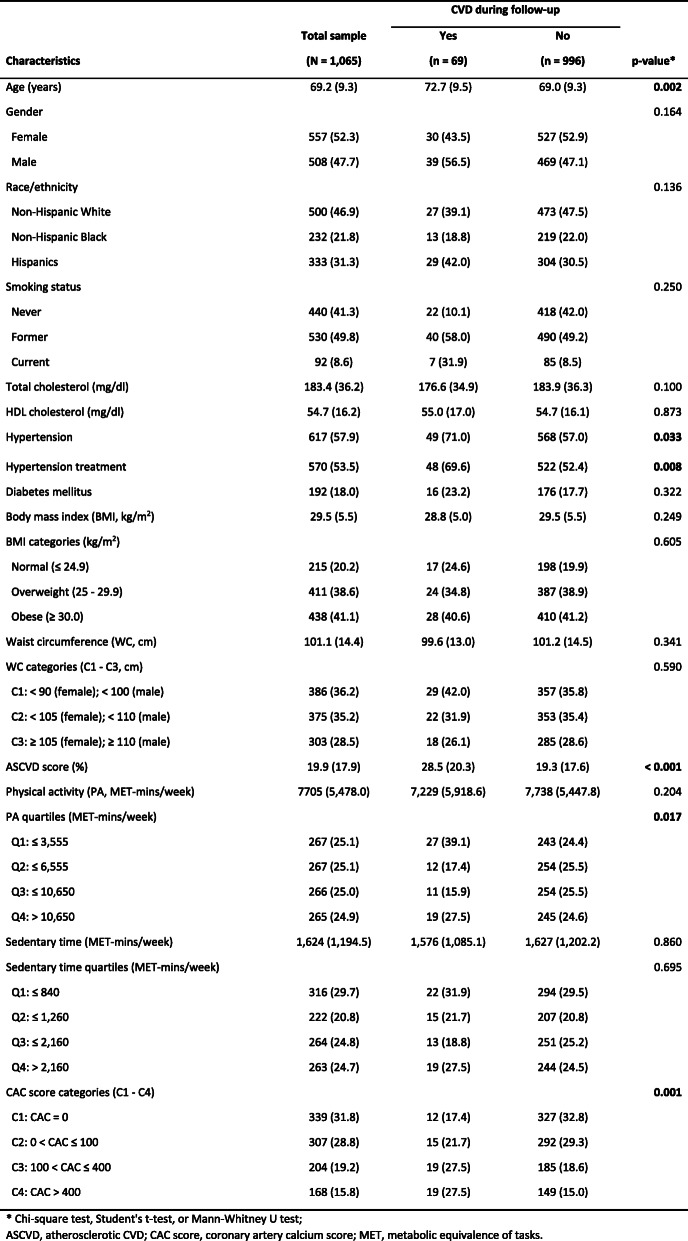
Baseline demographic, behavioral, and risk factor characteristics by CVD outcome during follow-up (mean, SD or N, %)

As shown in Table 2, several baseline summary measures were significantly different across the PA quartiles (PA Q1–4). From the PA Q1 to Q4, the mean ages of participants decreased from 72.6 years to 66.0 years, which indicated that older people were more likely to be physically inactive. A higher proportion of males (55.1%) than females (44.9%) were observed in PA Q1. Females were dominant (> 54.0%) in all other three higher quartiles (PA Q2–3), indicating that females were more likely to be physically active than males. Regarding the clinical characteristics, hypertension prevalence at baseline was higher in lower PA quartiles (i.e., PA Q1 and Q2; > 62.0%) than that in upper PA quartiles (i.e., PA Q3 and Q4; < 55%). Notably, the mean ASCVD score was highest in PA Q1 (25.9%) and lowest in the PA Q4 (15.8%). Similarly, the proportion of the participants with a high CAC score (i.e., greater than 400) decreased from 20.6% (PA Q1) to 14.4% (PA Q4).

**Table 2:**
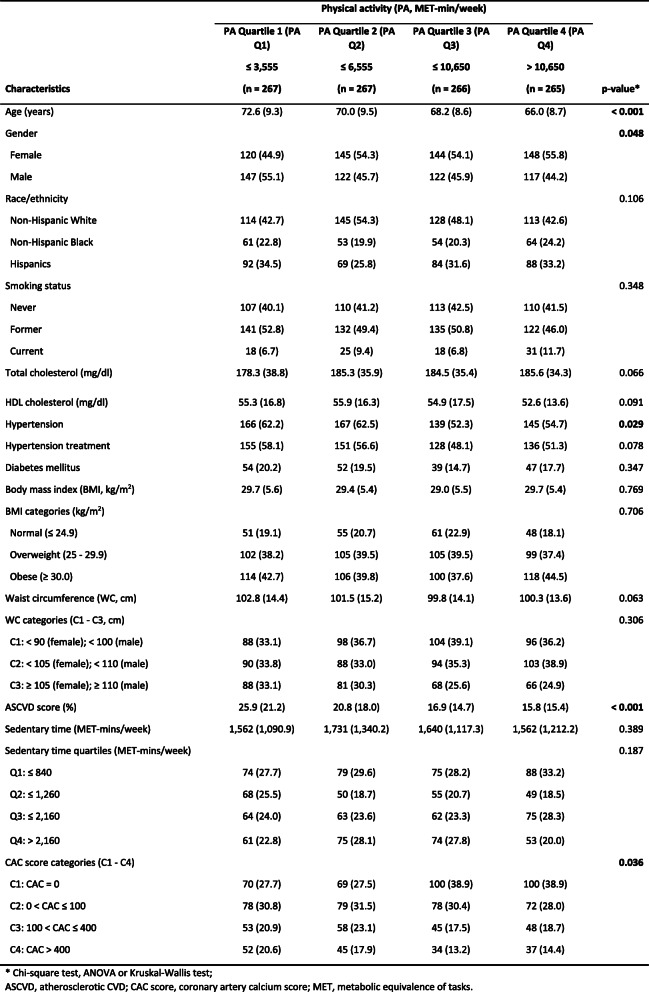
Baseline demographic, behavioral, and risk factor characteristics by physical activity intensity (mean, SD or N, %)

Table 3 displays the cumulative incidence of each CVD outcome across the PA quartiles. Besides overall CVD (6.5%), the most frequently identified outcome during the follow-up was hard CVD (3.9%), followed by stroke (2.3%). Across the PA quartiles, it is noticeable that more CVD (all) incident cases were observed in PA Q1 and PA Q4 with the cumulative incidences of about 10.0%. The middle two quartiles (i.e., PA Q2 and Q3) had less CVD (all) incident cases, with no more than 4.5% cumulative incidence. A similar “U” shape pattern was also observed for the CVD (hard) outcome. In contrast, stroke incidence did not have this pattern. The highest stroke cumulative incidence (5.2%) was observed in PA Q1. All the other three quartiles (PA Q2–4) had lower incidences (≤ 1.5%). Although the interesting “U” shape pattern was observed in this study, the similar pattern was not appreciable in cross-sectional studies using MESA dataset [37].

**Table 3:**
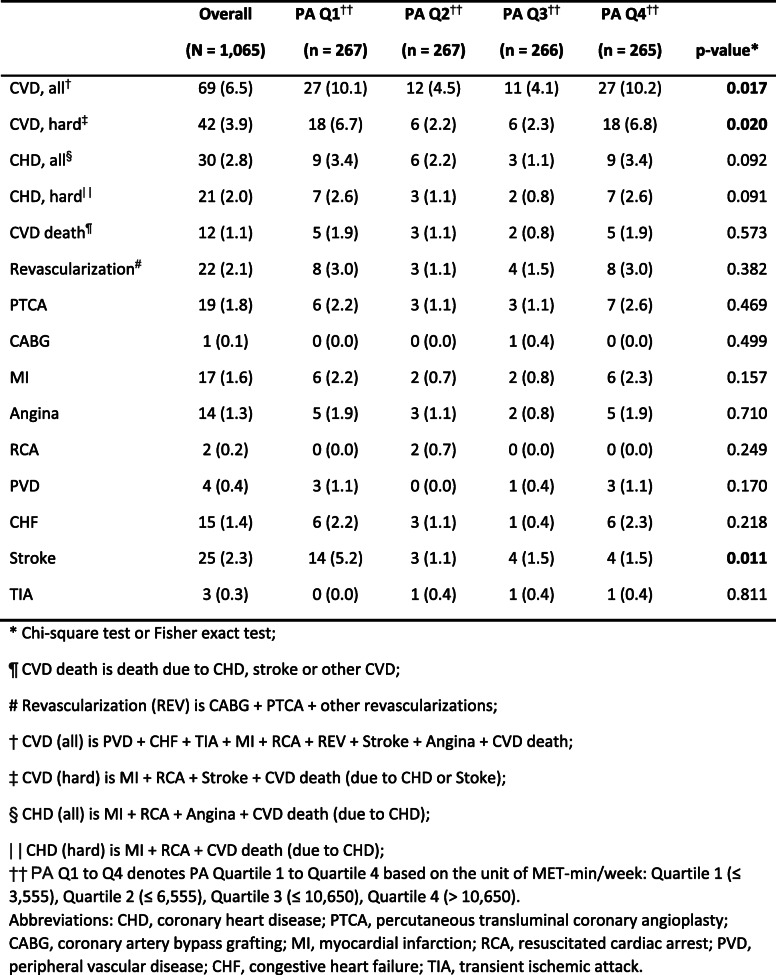
Total number of cases (N) and crude cumulative incidences (%) of CVD outcomes during follow-up by baseline physical activity (PA) intensity

### DNA methylation loci identification

**Fig. 1:**
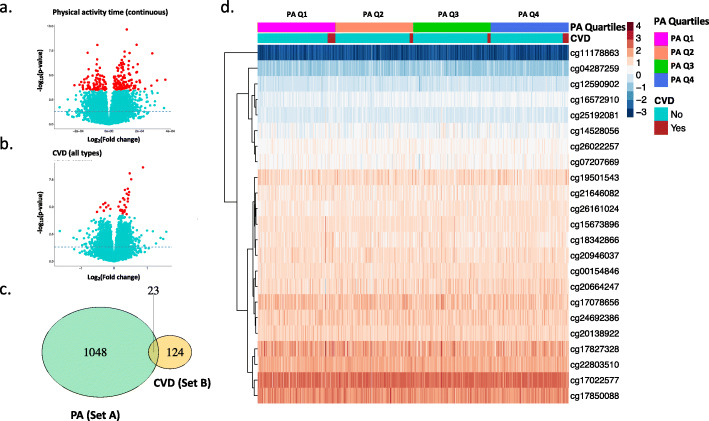
DNA methylation loci associated with PA and CVD. Representative volcano plots show the significant DNA methylation loci associated with continuous PA variable (**a**) and binary CVD outcome (**b**). DNA methylation loci were denoted as dots in red (significant loci) or in cyan (non-significant loci) according to the FDR cut-off value (0.05). The horizontal dashed line represents where *p*-value is 0.05. Venn diagram shows the overlapped 23 DNA methylation loci from two sets (**c**). The green and orange circles represent the set of DNA methylation loci associated with all PA variables (Set A) and all CVD variables (Set B), respectively. Heatmap (with M-value) shows the differential methylation level of the overlapped 23 loci in 1065 participants across PA quartiles and CVD status (**d**). Numbers beginning with “cg” are IlmnIDs

Representative volcano plots (Fig. 1a and b), Manhattan plots (Fig. S1), and Q-Q plots (Fig. S2) show the differentially methylated loci identified at the screening stage. The total number of candidate loci identified from each variable is presented in Supplementary Tables S3 and S4. As shown in the Venn diagram (Fig. 1c), a total number of 1048 loci were selected from the EWAS on PA variables (Set A), and 124 loci were from the EWAS on all the CVD variables (Set B). The overlapped 23 loci were then identified from the two sets. The detailed information of the overlapped 23 loci and corresponding gene names are listed in Supplementary Table S5. The heatmap shows the differential degree of methylation of the 23 loci grouped by four PA quartiles (PA Q1–4) and CVD (all) outcome (Fig. 1d).

### SEM model

The final SEM model was fitted among PA, 3 identified loci and CVD, along with factors such as waist circumference (WC), ASCVD score, CAC score, and ethnicity. 20 loci were excluded during the model selection due to non-significant correlations with CVD outcome. Similarly, sedentary time and BMI were also excluded due to a lack of correlation with other variables. The remaining three DNA methylation loci were cg17850088, cg04287259, and cg17078656, which corresponded to gene *VPS45*, *PIK3CD*, and *VPS13D*, respectively.

**Fig. 2:**
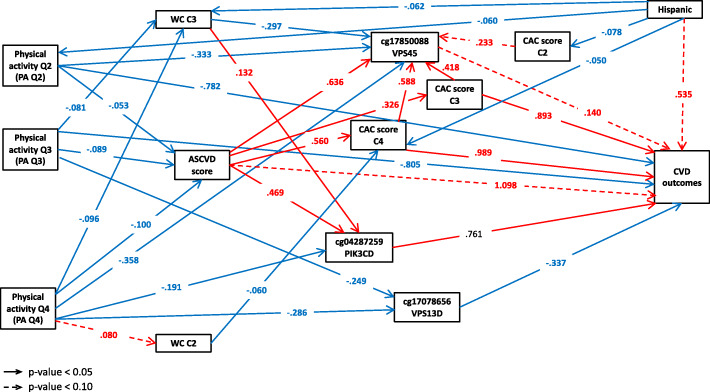
SEM model with standardized coefficient loads among PA, DNA methylation loci, CVD, and covariates. Each variable in the box represents a continuous variable or a quartile/category of a categorical variable. Numbers beginning with “cg” are IlmnIDs. *VPS45*, *PIK3CD*, and *VPS13D* are the corresponding gene names of the IlmnIDs. A red arrow represents a positive load (“stimulation”) from one variable to another, while a blue arrow represents a negative load (“inhibition”). Solid lines indicate the associations with a *p*-value < 0.05, whereas dashed lines for a *p*-value between 0.05 and 0.10. Abbreviations: ASCVD, atherosclerotic CVD; CAC, coronary artery calcium score; WC, waist circumference; Q2-Q4, quartile 2 to 4; C2-C4, category 2 to 4

Figure 2 shows the path diagram of the final SEM with estimated standardized regression coefficients (β). A positive load suggested a positive association with CVD incidence (i.e., a “stimulation” path), while a negative load suggested an inverse association (i.e., an “inhibition” path). Focusing on the correlations among PA, DNA methylation, and CVD, direct loads on CVD were found from PA quartile 2 (PA Q2 ⇒ CVD, β − 0.782), PA quartile 3 (PA Q3 ⇒ CVD, β − 0.805), and the three genes (*VPS45* ⇒ CVD [β 0.140], *PIK3CD* ⇒ CVD [β 0.761], and *VPS13D* ⇒ CVD [β − 0.337]). PA quartile 2 (PA Q2) and PA quartile 3 (PA Q3) showed negative loads on CVD (i.e., inhibition on CVD incidence), with the greatest absolute value in the association from PA quartile 3 (|β| = 0.805). Among the three genes, *VPS45* and *PIK3CD* had positive loads (“stimulation”) on CVD, indicating they were positively associated with CVD incidence. In contrast, gene *VPS13D* was inversely associated with CVD incidence (“inhibition”) due to a significant negative load. Located in the central part of the path diagram, the three genes, along with ASCVD score, CAC score, and WC, engaged as mediators bridging other variables to CVD through indirect paths. Counted from the total number of path arrows pointed to the mediators, gene *VPS45* (seven in total) was found to have the largest number of indirect paths mediating to CVD, followed by mediators such as gene *PIK3CD*, ASCVD score, and gene *VPS13D*.

The standardized total effect, direct path effect, and indirect path effects are summarized in Table 4. We calculated the total effects of PA on CVD from the sum of all direct and indirect path effects. For example, the total effect of PA Q2 → CVD (β = − 0.959 = [− 0.782] + [− 0.047] + [− 0.130]) can be decomposed into direct effect (PA Q2 ⇒ CVD, β − 0.782) and indirect paths through *VPS45* (PA Q2 ⇒ *VPS45* → CVD, β − 0.047) and ASCVD score (PA Q2 ⇒ ASCVD→CVD, β − 0.130). Similarly, PA Q3 and Q4 (vs PA Q1) each had multiple paths involving a significant indirect path through reducing the effect of ASCVD score (the variable with the largest total effect [|β| = 2.454] on CVD). However, different from PA Q2, PA Q3 and Q4 started to have indirect “stimulation” path to CVD incidence. The total effect of PA Q3 on CVD consisted of three “inhibition” paths, and one “stimulation” path through *VPS13D* (β = 0.084). The total effect of PA Q4 on CVD was the summation of five “inhibition” paths, and one “stimulation” indirect path through *VPS13D* (β = 0.096). Despite the existence of the “stimulation” paths, the total effects of all PA quartiles were negative, indicating inverse associations (“inhibition”) with CVD incidence. However, the overall association of PA Q4 with CVD was much weaker (β = − 0.355) than PA Q2 (β = − 0.959) and Q3 (β = − 0.944). In sensitivity analyses, the coefficients and significant paths between CVD and other variables were robust in all models described in the Methods section.

**Table 4:**
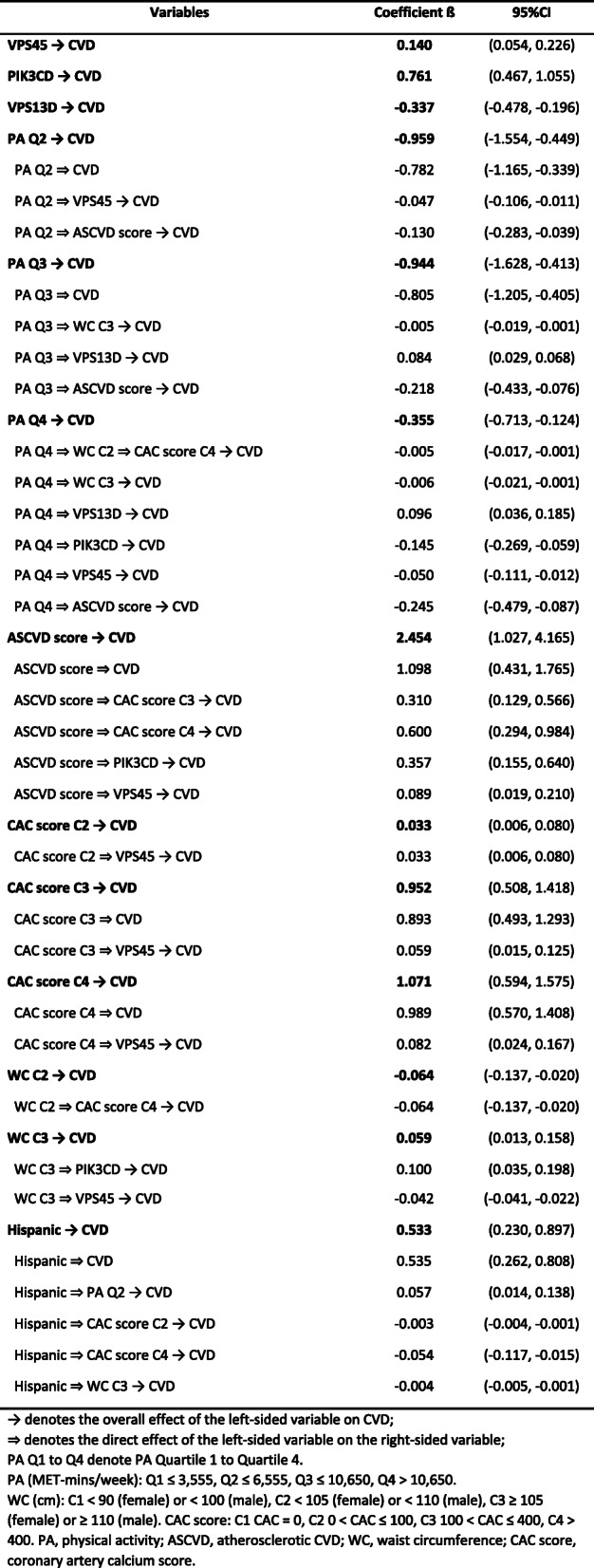
Overall coefficient loads on CVD in SEM model

## Discussion

The current study is the first to simultaneously examine the relationships among PA, DNA methylation, and CVD in a large multiethnic cohort with long-term exposure. The major finding of the present work is the identification of three DNA methylation loci (corresponding to genes *PIK3CD*, *VPS45*, and *VPS13D*) playing as mediators linking the association between PA and CVD. Although we used the screened significant loci to fit the SEM model, the *p*-values distribution of the screened loci in SEM complied uniform pattern. Results of the SEM depicted a network with both direct and indirect pathways showing how PA was associated with the reduced CVD incidence through different genes and/or other risk factors.

Consistent with most previous studies [38], we showed that the increase of habitual PA time was associated with decreased incidence of CVD, although not totally linear (strongest with PA Q2 and Q3, but relatively weaker with Q4). The magnitude of the association between CVD and PA Q2 (β = − 0.959) or Q3 (β = − 0.944) was equivalent to > 30% of the association between CVD and ASCVD score (β = 2.454). Such strong quantified associations highlighted the importance of being physically active in the reduction of CVD incidence. However, the highest level of PA quartile (PA Q4) was found to have the weakest protective effect on the cardiovascular system due to the greatest contribution of the “stimulation” path components (Fig. 2 and Table 4). The reduction of the inhibition effect of PA as the intensity approaches vigorous activity has been previously observed [39, 40]. Additionally, growing evidence has shown that vigorous activity can even acutely and transiently increase the risk of CVD in susceptible persons with congenital anomalies or existing cardiac diseases [41]. It is noted that in the present study we fitted models using total PA, which may be different from the studies focusing on vigorous PA or excessive exercise [20, 37]. The difference may explain why we observed harmful components (with overall protective effect) of heigh level PA, but not overall harmful effect reported in some previous studies [42]. Although the mechanism by which vigorous PA has harmful components or effect on the cardiovascular system is not well understood [43], we provided one promising biological mechanism – the epigenetic modification of gene *VPS13D* – based on the role as a mediator in the SEM model.

The biological functions of the identified genes in the SEM model were strongly related to the development of atherosclerosis. As the dominant cause of CVD, atherosclerosis is in fact a chronic inflammatory condition involving immune cells (e.g., macrophages, etc.) and pro-inflammatory cytokines [44]. The monocyte-derived macrophage migrates to lesions in the vessel and contributes to every phase of atherogenesis [45]. The initial atherosclerotic plaque is characterized by the accumulation of lipid-laden macrophages. The activated macrophages then secrete cytokines (e.g., TNF-α and IL-1), enzymes, and growth factors (e.g., PDGF), and contribute to the further development of atherosclerotic plaque [46]. As the key mediators in the SEM model, genes *VPS13D* and *PIK3CD* are both related to monocyte-macrophage differentiation [47]. The *VPS13D* (vacuolar protein sorting 13 homolog D) gene encodes a protein that plays an important role in autophagy [48]. The induction of autophagy is found to be essential for the survival and differentiation of monocytes to macrophages [49]. *PIK3CD* (phosphatidylinositol-4,5-bisphosphate 3-kinase [PIK3] catalytic subunit δ) encodes a subunit of the enzyme PIK3 which phosphorylates inositol and is involved in the immune response through the PIK3/AKT pathway [50]. The PIK3/AKT/mTOR pathway has been demonstrated to regulate monocyte-derived macrophage activation and polarization [47]. Furthermore, PIK3 was also found to limit the production of TNF-α in activated monocytes [51] and to regulate the activation of platelet [52]. Notably, the polymorphism of the genes *VPS13D* and *PIK3* were also identified to be associated with CVD in other cohorts [52, 53]. Gene *VPS45* (vacuolar protein sorting 45 homolog) is currently not well studied. The knowledge of *VPS45*-related disease in humans is limited to its association with congenital neutropenia [54]. Considering the important effect of *VPS45* identified in our SEM model, future biological studies on *VPS45* and its role in the development of CVD are promising.

Several limitations in this study are noted. First, the habitual PA time was collected through surveys at Exam 5 along with the collection of samples for epigenetics. Although the interviewers were well trained, recall bias may have been introduced because of self-reporting. Also, although the formation of PA habitus occurred prior to the collection of epigenetics information, the time order of PA habitus and epigenetic pattern formation was still not clear. Second, the time-varying variables are difficult to control in an observational study without a follow-up visit between baseline and endpoint. Third, a survivorship bias was introduced when we used MESA Exam 5 as a baseline and excluded the CVD prevalent cases. By Exam 5, the MESA study has continued for nearly 10 years since Exam 1. People who did not develop CVD during the last four visits were possibly those who were less likely to have CVD due to genetic or environmental factors. However, since the DNA methylation data were collected during Exam 5, the introduction of a survivorship bias is inevitable. Fourth, although the MESA study included Asian-American participants, this group was not included in the current study because they were not represented in the MESA Epigenomics and Transcriptomics Study. Thus, the interpretation of the results may not be generalizable to Asian-American populations. In addition, because of the potential heterogeneity [55] and misclassification [56] in the Hispanic population of the MESA cohort. The interpretation of the direct effect of Hispanics on CVD had limitation. Fifth, although two of the three identified genes in this study were also identified by other GWAS cohorts, the current study was the only study reported the association between CVD and the methylation of the genes *PIK3CD*, *VPS45*, and *VPS13D*. The possibility of false positive results should be noted due to the lack of replication. Finally, the effects of genes in this study were only indicative due to the lack of causal inference regarding both statistics and biology. The term “mediator” used in this study only defined the linking variable in SEM, without demonstration of causal mediation effects. Although it is reasonable to assume that DNA methylations mediate the effects of an exposure on a disease since DNA methylation is malleable to environmental changes [57, 58], causation assumptions in high-dimensional omics data are usually not justified without the application of approaches such as global test [59] and Mendelian randomization [60]. It is noteworthy that we interpreted the “stimulation” or “inhibition” effects of genes based on the positive or negative signs of the coefficients, and the assumption that DNA methylation commonly represses gene expression. However, growing evidences showed that DNA methylation can also increase gene expression in some instances [9]. Thus, the actual biological roles of identified genes should be further studied by mechanism research.

Interpreted with a few limitations in mind, the current study is still among the first to simultaneously examine the relationships among PA, DNA methylation, and CVD in a large multiethnic cohort with long-term exposure. The identified genes might have the potential to be therapeutic targets for future CVD prevention or to be involved in the modification of CVD risk score.

## Conclusion

Our study was among the first to simultaneously examine the complexity of physical activity (PA) and DNA methylation interaction in the development of (CVD) in a large cohort. The major finding of the present work is the identification of three DNA methylation loci (corresponding to genes *PIK3CD*, *VPS45*, and *VPS13D*) bridging the association between PA and CVD. The function of those three genes warrants further investigation in the pathogenesis of CVD, with potentially therapeutic or predictive implications for clinical care.

## Supplementary Information

**Additional file 1.**

## Data Availability

Illumina HumanMethylation450 (450 K) BeadChip data of the MESA Epigenomics and Transcriptomics Study (2010–2012) are publicly accessible through the GEO website with accession ID GSE56046. The website is: https://www.ncbi.nlm.nih.gov/geo/query/acc.cgi?acc=GSE56046

## Abbreviations

CVD: Cardiovascular disease
PA: Physical activity
MESA: Multi-Ethic Study of Atherosclerosis
SEM: Structural equation modeling
BMI: Body mass index
WC: Waist circumference
ASCVD: Atherosclerotic cardiovascular disease
CAC: Coronary artery calcium
MET: Metabolic equivalence of tasks

## References

[1] Roth GA, Mensah GA, Johnson CO, Addolorato G, Ammirati E, Baddour LM, Barengo NC, Beaton AZ, Benjamin EJ, Benziger CP (2020). Global burden of cardiovascular diseases and risk factors, 1990-2019: update from the GBD 2019 study. J Am Coll Cardiol.

[2] Stewart J, Manmathan G, Wilkinson P (2017). Primary prevention of cardiovascular disease: a review of contemporary guidance and literature. JRSM Cardiovasc Dis.

[3] Kathiresan S, Srivastava D (2012). Genetics of human cardiovascular disease. Cell.

[4] Eijsvogels TM, Molossi S, Lee DC, Emery MS, Thompson PD (2016). Exercise at the extremes: the amount of exercise to reduce cardiovascular events. J Am Coll Cardiol.

[5] Gleeson M, Bishop NC, Stensel DJ, Lindley MR, Mastana SS, Nimmo MA (2011). The anti-inflammatory effects of exercise: mechanisms and implications for the prevention and treatment of disease. Nat Rev Immunol.

[6] Grazioli E, Dimauro I, Mercatelli N, Wang G, Pitsiladis Y, Di Luigi L, Caporossi D (2017). Physical activity in the prevention of human diseases: role of epigenetic modifications. BMC Genomics.

[7] Devaux Y, Robinson EL (2021). Epigenetics in cardiovascular disease: Elsevier science.

[8] van der Harst P, de Windt LJ, Chambers JC (2017). Translational perspective on epigenetics in cardiovascular disease. J Am Coll Cardiol.

[9] Moore LD, Le T, Fan G (2013). DNA methylation and its basic function. Neuropsychopharmacology.

[10] Goulle JP, Guerbet M (2020). Recreational use of cannabis: from effects to harm. Epidemiological data. Bull Acad Natl Med.

[11] Meder B, Haas J, Sedaghat-Hamedani F, Kayvanpour E, Frese K, Lai A, Nietsch R, Scheiner C, Mester S, Bordalo DM (2017). Epigenome-wide association study identifies cardiac gene patterning and a novel class of biomarkers for heart failure. Circulation.

[12] Fernandez-Sanles A, Sayols-Baixeras S, Castro DEMM, Esteller M, Subirana I, Torres-Cuevas S, Perez-Fernandez S, Aslibekyan S, Marrugat J, Elosua R (2020). Physical activity and genome-wide DNA methylation: the REgistre GIroni del COR study. Med Sci Sports Exerc.

[13] Recchioni R, Marcheselli F, Antonicelli R, Mensa E, Lazzarini R, Procopio AD, Olivieri F (2017). Epigenetic effects of physical activity in elderly patients with cardiovascular disease. Exp Gerontol.

[14] Zhong J, Agha G, Baccarelli AA (2016). The role of DNA methylation in cardiovascular risk and disease: methodological aspects, study design, and data analysis for epidemiological studies. Circ Res.

[15] Antonogeorgos G, Panagiotakos DB, Pitsavos C, Papageorgiou C, Chrysohoou C, Papadimitriou GN, Stefanadis C (2012). Understanding the role of depression and anxiety on cardiovascular disease risk, using structural equation modeling; the mediating effect of the Mediterranean diet and physical activity: the ATTICA study. Ann Epidemiol.

[16] Bild DE, Bluemke DA, Burke GL, Detrano R, Diez Roux AV, Folsom AR, Greenland P, Jacob DR, Kronmal R, Liu K (2002). Multi-ethnic study of atherosclerosis: objectives and design. Am J Epidemiol.

[17] Liu Y, Ding J, Reynolds LM, Lohman K, Register TC, De La Fuente A, Howard TD, Hawkins GA, Cui W, Morris J (2013). Methylomics of gene expression in human monocytes. Hum Mol Genet.

[18] Chi GC, Liu Y, MacDonald JW, Barr RG, Donohue KM, Hensley MD, Hou L, McCall CE, Reynolds LM, Siscovick DS (2016). Long-term outdoor air pollution and DNA methylation in circulating monocytes: results from the multi-ethnic study of atherosclerosis (MESA). Environ Health.

[19] Ainsworth BE, Haskell WL, Herrmann SD, Meckes N, Bassett DR, Tudor-Locke C, Greer JL, Vezina J, Whitt-Glover MC, Leon AS (2011). 2011 compendium of physical activities: a second update of codes and MET values. Med Sci Sports Exerc.

[20] Joseph JJ, Echouffo-Tcheugui JB, Golden SH, Chen H, Jenny NS, Carnethon MR, Jacobs D, Burke GL, Vaidya D, Ouyang P (2016). Physical activity, sedentary behaviors and the incidence of type 2 diabetes mellitus: the multi-ethnic study of atherosclerosis (MESA). BMJ Open Diabetes Res Care.

[21] Goff DC, Lloyd-Jones DM, Bennett G, Coady S, D'Agostino RB, Gibbons R, Greenland P, Lackland DT, Levy D, O'Donnell CJ (2014). 2013 ACC/AHA guideline on the assessment of cardiovascular risk: a report of the American College of Cardiology/American Heart Association task force on practice guidelines. Circulation.

[22] Grundy SM, Stone NJ, Bailey AL, Beam C, Birtcher KK, Blumenthal RS, Braun LT, de Ferranti S, Faiella-Tommasino J, Forman DE (2019). 2018 AHA/ACC/AACVPR/AAPA/ABC/ACPM/ADA/AGS/APhA/ASPC/NLA/PCNA guideline on the Management of Blood Cholesterol: a report of the American College of Cardiology/American Heart Association task force on clinical practice guidelines. Circulation.

[23] Greenland P, Blaha MJ, Budoff MJ, Erbel R, Watson KE (2018). Coronary calcium score and cardiovascular risk. J Am Coll Cardiol.

[24] Zhu S, Heymsfield SB, Toyoshima H, Wang Z, Pietrobelli A, Heshka S (2005). Race-ethnicity-specific waist circumference cutoffs for identifying cardiovascular disease risk factors. Am J Clin Nutr.

[25] McClelland RL, Chung H, Detrano R, Post W, Kronmal RA (2006). Distribution of coronary artery calcium by race, gender, and age: results from the multi-ethnic study of atherosclerosis (MESA). Circulation.

[26] Ross R, Neeland IJ, Yamashita S, Shai I, Seidell J, Magni P, Santos RD, Arsenault B, Cuevas A, Hu FB (2020). Waist circumference as a vital sign in clinical practice: a consensus statement from the IAS and ICCR working group on visceral obesity. Nat Rev Endocrinol.

[27] Neves PO, Andrade J, Moncao H (2017). Coronary artery calcium score: current status. Radiol Bras.

[28] Budoff MJ, Nasir K, McClelland RL, Detrano R, Wong N, Blumenthal RS, Kondos G, Kronmal RA (2009). Coronary calcium predicts events better with absolute calcium scores than age-sex-race/ethnicity percentiles: MESA (multi-ethnic study of atherosclerosis). J Am Coll Cardiol.

[29] Sundar IK, Yin Q, Baier BS, Yan L, Mazur W, Li D, Susiarjo M, Rahman I (2017). DNA methylation profiling in peripheral lung tissues of smokers and patients with COPD. Clin Epigenetics.

[30] Ritchie ME, Phipson B, Wu D, Hu Y, Law CW, Shi W (2015). Smyth GK: limma powers differential expression analyses for RNA-sequencing and microarray studies. Nucleic Acids Res.

[31] Bolstad B (2019). preprocessCore: A collection of pre-processing functions.

[32] Li D, Xie Z, Pape ML, Dye T (2015). An evaluation of statistical methods for DNA methylation microarray data analysis. BMC Bioinformatics.

[33] Li D, Xie Z, Zand M, Fogg T, Dye T (2017). Bon-EV: an improved multiple testing procedure for controlling false discovery rates. BMC Bioinformatics.

[34] Wickham H (2016). ggplot2: elegant graphics for data analysis.

[35] Kolde R (2019). pheatmap: Pretty Heatmaps.

[36] Muthén B (1984). A general structural equation model with dichotomous, ordered categorical, and continuous latent variable indicators. Psychometrika.

[37] Bertoni AG, Whitt-Glover MC, Chung H, Le KY, Barr RG, Mahesh M, Jenny NS, Burke GL, Jacobs DR (2009). The association between physical activity and subclinical atherosclerosis: the multi-ethnic study of atherosclerosis. Am J Epidemiol.

[38] Shiroma EJ, Lee IM (2010). Physical activity and cardiovascular health: lessons learned from epidemiological studies across age, gender, and race/ethnicity. Circulation.

[39] Lee DC, Pate RR, Lavie CJ, Sui X, Church TS, Blair SN (2014). Leisure-time running reduces all-cause and cardiovascular mortality risk. J Am Coll Cardiol.

[40] Mohamadzade B, Hashmi RM, Simorangkir R, Gharaei R, Ur Rehman S, Abbasi QH. Recent Advances in Fabrication Methods for Flexible Antennas in Wearable Devices: State of the Art. Sensors (Basel). 2019;19(10). 10.3390/s19102312 PMC6567739 31109158

[41] Buttar HS, Li T, Ravi N (2005). Prevention of cardiovascular diseases: role of exercise, dietary interventions, obesity and smoking cessation. Exp Clin Cardiol.

[42] O'Keefe JH, Patil HR, Lavie CJ, Magalski A, Vogel RA, McCullough PA (2012). Potential adverse cardiovascular effects from excessive endurance exercise. Mayo Clin Proc.

[43] Thompson PD, Franklin BA, Balady GJ, Blair SN, Corrado D, Estes NA, Fulton JE, Gordon NF, Haskell WL, Link MS (2007). Exercise and acute cardiovascular events placing the risks into perspective: a scientific statement from the American Heart Association Council on nutrition, physical activity, and metabolism and the council on clinical cardiology. Circulation.

[44] Frostegard J (2013). Immunity, atherosclerosis and cardiovascular disease. BMC Med.

[45] Ross R (1999). Atherosclerosis--an inflammatory disease. N Engl J Med.

[46] Alexander MR, Owens GK (2012). Epigenetic control of smooth muscle cell differentiation and phenotypic switching in vascular development and disease. Annu Rev Physiol.

[47] Vergadi E, Ieronymaki E, Lyroni K, Vaporidi K, Tsatsanis C (2017). Akt signaling pathway in macrophage activation and M1/M2 polarization. J Immunol.

[48] Wang Z, Zhang H (2018). Mitophagy: Vps13D couples mitochondrial fission and Autophagic clearance. Curr Biol.

[49] Zhang Y, Morgan MJ, Chen K, Choksi S, Liu ZG (2012). Induction of autophagy is essential for monocyte-macrophage differentiation. Blood.

[50] Weichhart T, Saemann MD (2008). The PI3K/Akt/mTOR pathway in innate immune cells: emerging therapeutic applications. Ann Rheum Dis.

[51] Kramer PR, Winger V, Reuben J (2009). PI3K limits TNF-alpha production in CD16-activated monocytes. Eur J Immunol.

[52] Durrant TN, Hers I (2020). PI3K inhibitors in thrombosis and cardiovascular disease. Clin Transl Med.

[53] Koriyama H, Nakagami H, Katsuya T, Sugimoto K, Yamashita H, Takami Y, Maeda S, Kubo M, Takahashi A, Nakamura Y (2010). Identification of evidence suggestive of an association with peripheral arterial disease at the OSBPL10 locus by genome-wide investigation in the Japanese population. J Atheroscler Thromb.

[54] Stepensky P, Saada A, Cowan M, Tabib A, Fischer U, Berkun Y, Saleh H, Simanovsky N, Kogot-Levin A, Weintraub M (2013). The Thr224Asn mutation in the VPS45 gene is associated with the congenital neutropenia and primary myelofibrosis of infancy. Blood.

[55] Manichaikul A, Palmas W, Rodriguez CJ, Peralta CA, Divers J, Guo X, Chen WM, Wong Q, Williams K, Kerr KF (2012). Population structure of Hispanics in the United States: the multi-ethnic study of atherosclerosis. PLoS Genet.

[56] Divers J, Redden DT, Rice KM, Vaughan LK, Padilla MA, Allison DB, Bluemke DA, Young HJ, Arnett DK (2011). Comparing self-reported ethnicity to genetic background measures in the context of the multi-ethnic study of atherosclerosis (MESA). BMC Genet.

[57] Fasanelli F, Baglietto L, Ponzi E, Guida F, Campanella G, Johansson M, Grankvist K, Johansson M, Assumma MB, Naccarati A (2015). Hypomethylation of smoking-related genes is associated with future lung cancer in four prospective cohorts. Nat Commun.

[58] Sandanger TM, Nost TH, Guida F, Rylander C, Campanella G, Muller DC, van Dongen J, Boomsma DI, Johansson M, Vineis P (2018). DNA methylation and associated gene expression in blood prior to lung cancer diagnosis in the Norwegian women and Cancer cohort. Sci Rep.

[59] Djordjilovic V, Page CM, Gran JM, Nost TH, Sandanger TM, Veierod MB, Thoresen M (2019). Global test for high-dimensional mediation: testing groups of potential mediators. Stat Med.

[60] Richmond RC, Hemani G, Tilling K, Davey Smith G, Relton CL (2016). Challenges and novel approaches for investigating molecular mediation. Hum Mol Genet.

