# Supplementary material for: Role of DNA methylation on the association between physical activity and cardiovascular diseases: results from the longitudinal multi-ethnic study of atherosclerosis (MESA) cohort
Source: BMC Genomics. 2021 Nov 3;22:790. doi: 10.1186/s12864-021-08108-w (PMC8567593; doi:10.1186/s12864-021-08108-w)
Supplement: Supplementary file 1 — Additional file 1. [file 12864_2021_8108_MOESM1_ESM.docx]

**Supplementary materials content**

**Role of DNA Methylation on the Association between Physical Activity and Cardiovascular Diseases: Results from the Longitudinal Multi-Ethnic Study of Atherosclerosis (MESA) Cohort**

Shi. et al.

**Supplementary Tables & Figures**

**Table S1.** Description of physical activity (PA) and sedentary behavior variables

**Table S2.** Description of CVD outcomes

**Table S3.** Total number of identified DNA methylation loci by each physical activity (PA) variable

**Table S4.** Total number of identified DNA methylation loci by each CVD outcome

**Table S5.** Information of the overlapped 23 genes

**Figure S1.** Manhattan plots

**Figure S2.** Q-Q plots

**Supplementary Raw Datasets**

Results of univariate EWAS.zip

| **Table S1. Description of physical activity (PA) and sedentary behavior variables*** | | | | | | | |
| --- | --- | --- | --- | --- | --- | --- | --- |
| **Variables** | **Description** | **MET** | **Continuous variables** | | **Categorical variables** | |  |
|  |  |  | **mins/week** | **MET-mins/week** | **mins/week** | **MET-mins/week** |  |
| Total PA | light + moderate + vigorous PA | MET > 1.5 | √ | √ | √ | √ |  |
| MVPA | moderate + vigorous PA | MET ≥ 3 | √ | √ | √ | √ |  |
| Light PA^¶^ |  | 1.5 < MET < 3 | √ | √ | √ | √ |  |
| Moderate PA^#^ |  | 3 ≤ MET < 6 | √ | √ | √ | √ |  |
| Vigorous PA^†^ |  | MET ≥ 6 | √ | √ | √ | √ |  |
| Exercise PA^‡^ | intentional exercise | 3.5 ≤ MET ≤ 7 | √ | √ | √ | √ |  |
| Sedentary^§^ | sedentary behavior | MET ≤ 1.5 | √ | √ | √ | √ |  |

* To increase the screening power, a total of 28 PA variables were tested separately for screening associated DNA methylation loci. The 28 PA variables are the seven combinations (light PA, moderate PA, vigorous PA, MVPA, exercise PA, total PA, and sedentary time) as both continuous and categorical variables (7*2) in both min/week and MET-mins/week units (7*2*2 = 28).

Hours of total PA and sedentary behavior per day were calculated for each participant. Those who reported > 24 hours or = 0 hour were excluded.

¶ Light PA includes light household chores (cooking, dishes, ironing, straightening up, laundry, shopping [MET 2.5]), care of others (bathing, feeding, changing diapers, playing with child [MET 2.5]), and work standing (MET 2.5).

# Moderate PA includes moderate house chores (heavy cleaning, scrubbing, mopping, home repairs, washing car, vacuuming [MET 4.0]), moderate yard work (weeding, mowing, raking, cleaning garage, sweeping [MET 4.0]), moderate care of others (lifting, carrying, pushing wheelchair [MET 4.0]), moderate walking (MET 3.0), moderate walking for exercise (MET 3.5), moderate dancing (MET 5.0), moderate individual activities (golf, bowling, yoga, Tai Chi [MET 3.5]), moderate conditioning activities (low impact aerobics, slow bicycling, rowing, leisurely swimming, health club machines [MET 5.5]), and moderate work (MET 3.0).

† Vigorous PA includes vigorous yard work (digging dirt, shoveling, mending fences, chopping wood [MET 6.5]), vigorous team sports (softball, volleyball, basketball, soccer [MET 7.0]), vigorous dual sports (tennis, racquetball, paddleball [MET 7.0]), vigorous conditioning activities (high impact aerobics, fast bicycling, running, jogging, fast swimming, judo, kickboxing, karate [MET 7.0]), and vigorous work (MET 7.0).

‡ Exercise PA includes moderate walking for exercise (MET 3.5), moderate dancing (MET 5.0), moderate individual activities (golf, bowling, yoga, Tai Chi [MET 3.5]), moderate conditioning activities (low impact aerobics, slow bicycling, rowing, leisurely swimming, health club machines [MET 5.5]), vigorous team sports (softball, volleyball, basketball, soccer [MET 7.0]), vigorous dual sports (tennis, racquetball, paddleball [MET 7.0]), and vigorous conditioning activities (high impact aerobics, fast bicycling, running, jogging, fast swimming, judo, kickboxing, karate [MET 7.0]).

§ Sedentary behavior includes watching TV (MET 1.0), reading (MET 1.5), and sitting (MET 1.5).

| **Table S2. Description of CVD outcomes** | | |
| --- | --- | --- |
| **Outcomes** | **Abbreviation** | **Description** |
| Cardiovascular disease (CVD), all |  | PVD + CHF + TIA + STRK + ANG + MI + REVa + RCA + CVDDTH |
| Cardiovascular disease (CVD), hard |  | STRK + MI + RCA + CVDDTH (due to CHD or STRK) |
| Coronary heart disease (CHD), all |  | ANG + MI + RCA + CVDDTH (due to CHD) |
| Coronary heart disease (CHD), hard |  | MI + RCA + CVDDTH (due to CHD) |
| Resuscitated cardiac arrest | RCA |  |
| CVD death | CVDDTH | death due to CHD, STRK, or other CVD |
| Revascularization, all | REVa | CABG + PTCA + REVo |
| Revascularization | REV | CABG + PTCA |
| Coronary artery bypass graft | CABG |  |
| Percutaneous transluminal coronary angioplasty | PTCA | PTCA, coronary stent, or coronary atherectomy |
| Other revascularizations | REVo |  |
| Myocardial infarction | MI |  |
| Angina pectoris | ANG |  |
| Congestive heart failure | CHF |  |
| Peripheral vascular disease | PVD |  |
| Stroke | STRK |  |
| Transient ischemic attack | TIA |  |

MESA assessed 11 individual cardiovascular events, including myocardial infarction (MI), angina pectoris (ANG), percutaneous transluminal coronary angioplasty (PTCA), coronary artery bypass graft (CABG), other revascularization, congestive heart failure (CHF), peripheral vascular disease (PVD), non-hemorrhagic stroke, transient ischemic attack (TIA), resuscitated cardiac arrest (RCA), and cardiovascular disease death. Six additional CVD outcomes were then created based on various combinations of individual cardiovascular events, including coronary heart disease (CHD) (all types), CHD (hard type), revascularization (PTCA + CABG), revascularization (all types), CVD (hard type), CVD (all types).

| **Table S3. Total number of identified DNA methylation loci by each physical activity (PA) variable** | | | | |
| --- | --- | --- | --- | --- |
| **Variables** | **Continuous variables** | | **Categorical variables** | |
|  | **mins/week** | **MET-mins/week** | **mins/week** | **MET-mins/week** |
| Total PA | 218 | 44 | 56 | 41 |
| MVPA | 2 | 2 | 3 | 0 |
| Light PA | 574 | 387 | 574 | 387 |
| Moderate PA | 1 | 0 | 0 | 0 |
| Vigorous PA | 51 | 376 | 49 | 379 |
| Exercise PA | 0 | 0 | 0 | 1 |
| Sedentary time | 0 | 0 | 0 | 0 |

| **Table S4. Total number of identified DNA methylation loci by each CVD outcome** | | |
| --- | --- | --- |
| **Outcomes** | **Prevalent cases related** | **Incident cases related** |
| Cardiovascular disease (CVD), all | 34 | 20 |
| Cardiovascular disease (CVD), hard | 10 | 0 |
| Coronary heart disease (CHD), all | 2 | 0 |
| Coronary heart disease (CHD), hard | 0 | 0 |
| Resuscitated cardiac arrest | 0 | 14 |
| CVD death | N/A | 2 |
| Revascularization, all | 0 | 1 |
| Revascularization | 1 | 2 |
| Coronary artery bypass graft | 25 | 20 |
| Percutaneous transluminal coronary angioplasty | 0 | 0 |
| Other revascularizations | 1 | 7 |
| Myocardial infarction | 1 | 0 |
| Angina pectoris | 1 | 0 |
| Congestive heart failure | 3 | 2 |
| Peripheral vascular disease | 19 | 9 |
| Stroke | 12 | 0 |
| Transient ischemic attack | 2 | 6 |

| **Table S5. Information of the overlapped 23 genes** | | | | |
| --- | --- | --- | --- | --- |
| **IlmnID** | **Gene** | **Strand** | **Location** | **Source Sequence** |
| cg20664247 | PEX10 | R | N_Shore | CGGATGAGGGGTTCAGCCCAAGGTGCTCAACCCAATCAGAGGGCAGCCTC |
| cg17078656 | VPS13D | F |  | ACTGATCCTATGCTGACAAACTGAATGAATGTGAACTGCTGCCACTTCCG |
| cg24692386 | CROCC | F |  | TCTCCAGGCCTGGAGCTGGAGTACCCGTCCTCAGCACTGCCAGCAGAGCG |
| cg26022257 |  | R |  | CGCAAAAGTGCGCCCTCTACTGGAGGGGTGAAGAAACCTCATCGTTACAG |
| cg21646082 | CCDC21 | R | N_Shelf | AGGGCTGATTAGGGAACTGTGTCCTACCCACACTGGCATGTTGGATTACG |
| cg26161024 | MAP3K6 | F | S_Shore | AGGCTCAGATGCCAAGTCCCACTCCAGGACTGAAGGCTTTCGGGCAAGCG |
| cg17827328 | STK40 | F |  | CCTTGGTCCTGGGAGTGTAGTCGAGGGGTCGCACCTCCCTGCATACGCCG |
| cg25192081 | JAK1 | R |  | CGCAGGGATGTGTGAGTGACACTCAAGGACAAGAGCAATTCTCAGCTCCT |
| cg18342866 |  | R | N_Shelf | CATTGAGGTTCTCCTCCTGTTGGCTCAAGGGACTTTTCTGCTTTTTATCG |
| cg17850088 | VPS45 | R | N_Shelf | CGCTGTCTCCTCAGCGCTCCTCAGTGAGCTTGACACTACACGCACATAAA |
| cg04287259 | PIK3CD | F |  | CGGTGATGATGTGTGGTCTATTTGTAAATTGAGTGCAGCACATCTAATAT |
| cg00154846 | C1orf86 | F | S_Shelf | CGGTCCTGTTTCTGAACCCCATCCCAGCCATGGCCTGTGTCTGGGCCAGC |
| cg17022577 | WNT4 | R | S_Shelf | TCGTCCCTCGCCTGAATGGATGTTTTTATCCTCCTAAGAACTGTGCCTCG |
| cg20138922 | EPHB2 | R |  | CGCCTGGCCTCTTGCCCACTCCAGCTGAGCCACACTGACCTCTTGGCTGC |
| cg19501543 | ZMYM4 | R |  | TGGCTCCAGCATCTGCTTCTGGTGAGCGCTTCAGGAAGCTTCCAATCACG |
| cg12590902 | ERI3 | R |  | CGTGTAGCTAGACAGAATTGAATTCTAAGACCGTGTAGTTTCCAAAAGAG |
| cg22803510 | FNBP1L | F |  | CGCCTTTAGGGCATTTTGTTATTTCCGCTGAATCATTAGTTATTAGGATA |
| cg20946037 |  | R |  | CGCCACAGGGAAAAGAAGATATAAGACACAGAATGCTCCTTGCCTGGCTA |
| cg07207669 | EFNA1 | R | S_Shore | CGCCCCAGCACTCATGAGAACTTGTGAAAACTTACCTTGTCAAATGTGCA |
| cg14528056 | GBAP1 | R | N_Shelf | TTCCTGTTTCTTCAAAATCTCAAAGCTCTTCATTCAGCCAGGCATGGTCG |
| cg11178863 |  | R | Island | CGGCTCGGCCAAGCTCGTGTTCCTCGACCTCAGCTACAACAACTTGACCC |
| cg16572910 |  | F |  | CGCCTAGATGGAGCTATGGAGAATAAACAGAGCAGAAGTGTAGTGGTCTG |
| cg15673896 | ADORA3 | R |  | CGGATCTTGCTGGCTCACCTGTCCCTGTGGAGGTTCCCCTGGGAAGGCAA |

**Figure S1. Manhattan plots**

**Continuous Total Physical activity**

**a.**

**b.**

**CVD (all types)**

**Figure S2. Q-Q plots**

**Continuous Total Physical activity**

**a.**

**λ** = 1.058

**b.**

**CVD (all types)**

**λ** = 0.972
